# Multiple evanescent white dot syndrome (MEWDS) and inactivated COVID-19 vaccination

**DOI:** 10.5935/0004-2749.2022-0112

**Published:** 2025-08-21

**Authors:** Won Sriwijitalai, Viroj Wiwanitkit

**Affiliations:** 1 Private Academic Consultant, Dimapur, India; 2 Honorary professor, Dr. DY Patil University, Pune, India

Dear Editor,

We would like to share our viewpoints on the article entitled “Multiple evanescent white dot syndrome (MEWDS) following inactivated COVID-19 vaccination (Sinovac-CoronaVac)^(1)^” by Tomishige et al.^(1)^ The authors presented a relevant case and discussed about the interplay of COVID-19 immunization and the case. We agree, based on the accumulated recorded data from post-vaccination adverse effect surveillance system, that the COVID-19 vaccination can result in a variety of clinical issues, including eye disorders. The current case may or may not, however, be related to the COVID-19 vaccination. For instance, there is no information about the patient’s immunological or ocular condition prior to vaccination. Conclusions about any interaction are therefore difficult to reach. It is important to keep in mind that the recurrence could be caused by a past medical condition or a concurrent medical disorder. After vaccination, for example, the vaccine recipient may get an arbovirus infection^(2)^. The present case could hence be that of a rare but probable cause of MEWDS from arbovirus infection^(3)^.
